# Comparison of Ceftolozane–Tazobactam Versus Meropenem Regimens in Treating Bloodstream Infections Caused by Extended-Spectrum β-Lactamase-Producing *Enterobacterales*: Real-World Data from a Greek Tertiary Center

**DOI:** 10.3390/jcm15166414

**Published:** 2026-08-19

**Authors:** Vasileios Petrakis, Petros Rafailidis, Andreas G. Tsantes, Dimitrios Themelidis, Nikoleta Babaka, Petros Ouzounakis, Georgios Lazaridis, Aikaterini Taniou, Alexandra Sarantopoulou, Dimitrios Papazoglou, Maria Panopoulou, Periklis Panagopoulos

**Affiliations:** 1 Department of Infectious Diseases, 2nd University Department of Internal Medicine, University General Hospital of Alexandroupolis, Democritus University Thrace, 68100 Alexandroupolis, Greece; prafaili@med.duth.gr (P.R.); mpampakanikoleta@gmail.com (N.B.); dpapazog@med.duth.gr (D.P.); ppanago@med.duth.gr (P.P.); 2 Laboratory of Haematology and Blood Bank Unit, “Attiko” Hospital, School of Medicine, National and Kapodistrian University of Athens, 12462 Athens, Greece; andreas.tsantes@yahoo.com; 3 Microbiology Department, “Saint Savvas” Oncology Hospital, 11522 Athens, Greece; 4 University Laboratory Department, University General Hospital of Alexandroupolis, Democritus University Thrace, 68100 Alexandroupolis, Greece; dthemelidis@gmail.com (D.T.); mpanopou@med.duth.gr (M.P.); 5 Infection Control Committee, University General Hospital of Alexandroupolis, Democritus University Thrace, 68100 Alexandroupolis, Greece; peterouzounakis@gmail.com (P.O.); glazarid@icloud.com (G.L.); 6 Faculty of Medicine, Democritus University Thrace, 68100 Alexandroupolis, Greece; ktaniou@gmail.com (A.T.); or alexsara7@med.duth.gr (A.S.)

**Keywords:** ceftolozane–tazobactam, meropenem, bloodstream infections, ESBL-producing *Enterobacterales*, carbapenem-sparing therapy, antimicrobial stewardship, Greece, real-world evidence

## Abstract

**Background/Objectives**: The rise of extended-spectrum β-lactamase (ESBL)-producing Enterobacterales has led to an increased carbapenem use, raising concerns regarding selection pressure for carbapenem-resistant organisms. Ceftolozane–tazobactam (C/T) is a potential effective carbapenem-sparing alternative. This single-centre retrospective study evaluated the clinical effectiveness and mortality predictors of ceftolozane–tazobactam versus meropenem as definitive targeted therapy for ESBL-producing *Enterobacterales* bloodstream infections (BSIs). **Methods**: We conducted a single-center retrospective analysis of adult hospitalized patients between January 2022 and February 2024 who presented with BSIs caused by ESBL-producing *Enterobacterales*. Patients (*N* = 185) were included if they received either C/T (*n* = 73) or optimized high-dose meropenem (*n* = 112) for at least 48 h. The primary clinical endpoint was all-cause 30-day mortality. Secondary endpoints included clinical success (cure), in-hospital mortality, treatment duration, microbiological eradication, and infection recurrence rates. A multivariable logistic regression model was executed to determine independent predictors of 30-day mortality. **Results**: *Escherichia coli* (54.1%) and *Klebsiella pneumoniae* (35.1%) were the primary pathogens. The raw clinical success rate was higher with C/T than meropenem (83.6% vs. 71.6%, *p* = 0.078). Unadjusted 30-day mortality was 12.3% for C/T and 19.6% for meropenem (*p* = 0.342). Zero recurrences occurred with C/T compared to an 8.0% recurrence rate with meropenem (0/73 [0.0%] in C/T vs. 9/112 [8.0%] in meropenem, *p* = 0.015). In the multivariable logistic regression analysis, definitive targeted treatment with C/T was independently associated with lower odds of all-cause 30-day mortality (Adjusted Odds Ratio [aOR] 0.60; 95% Confidence Interval [CI] 0.33–0.92; *p* = 0.022). Conversely, independent clinical mortality risks included male gender (*p* = 0.027), baseline SOFA score (*p* = 0.001), septic shock (*p* = 0.001), and an unknown primary infection source (*p* = 0.001). **Conclusions**: In this single-center retrospective observational cohort, definitive targeted therapy with ceftolozane–tazobactam was associated with favorable clinical success and lower adjusted 30-day mortality compared to meropenem in patients with ESBL *Enterobacterales* BSIs. These observational data support further prospective evaluation of C/T as a potential carbapenem-sparing option. Prospective randomized controlled trials are required to confirm these findings before clinical practice algorithms are modified.

## 1. Introduction

The increasing prevalence and global dissemination of multidrug-resistant (MDR) Gram-negative bacilli represent one major global public health challenge facing modern healthcare systems and clinical infectious disease networks [1]. Among these threats, the structural and epidemiological rise of *Enterobacterales* species that produce extended-spectrum β-lactamases (ESBLs)—primarily driven by the plasmid-mediated acquisition of enzymes such as the CTX-M, SHV, and TEM families—has substantially reduced the therapeutic utility of traditional first-line oxyimino-β-lactams, including third- and fourth-generation cephalosporins and monobactams [2]. Historically, the identification of an ESBL-producing phenotype in serious invasive pathogens, including *Escherichia coli*, *Klebsiella pneumoniae*, *Klebsiella oxytoca*, and *Proteus mirabilis*, automatically channelled clinical protocols toward the unconstrained administration of carbapenems, which have long been hailed as the standard of care for these infections, supported by randomized clinical trial evidence such as the MERINO study, which confirmed the clinical superiority of meropenem over piperacillin–tazobactam for ESBL bacteremia [3,4,5]. Consequently, current management guidelines from the Infectious Diseases Society of America (IDSA) and the European Society of Clinical Microbiology and Infectious Diseases (ESCMID) maintain carbapenems as the preferred first-line therapy for severe ESBL bloodstream infections (BSIs) [6,7].

However, this systemic clinical reliance on carbapenems has contributed to increasing antimicrobial selection pressure. The massive, sustained consumption of agents like meropenem, imipenem–cilastatin, and ertapenem has exerted substantial selective pressure on hospital-wide microbial flora [3,4]. This pressure has accelerated the selection, amplification, and horizontal gene transmission of secondary, more dangerous resistance mechanisms: specifically, serine- and metallo-β-lactamase carbapenemases [3,4,5,6,7]. The subsequent proliferation of carbapenem-resistant *Enterobacterales* (CRE) strains producing *Klebsiella pneumoniae* carbapenemase (KPC), oxacillinase-48 (OXA-48)-like, and New Delhi metallo-β-lactamase (NDM) enzymes has effectively exhausted our clinical armamentarium, frequently stranding clinicians with last-line therapeutic options such as colistin, polymyxin B, or aminoglycosides [3,4,5,6,7].

This precarious clinical paradigm is exceptionally pronounced within the specific epidemiological landscape of the Mediterranean basin, with Greece serving as one of the regions with a high burden of antimicrobial resistance [8]. Local surveillance registries and comprehensive multi-national networks, such as the Study for Monitoring Antimicrobial Resistance Trends (SMART), have long highlighted that Greece consistently records some of the highest baseline percentages of cephalosporin and carbapenem resistance across continental Europe [9]. In Greek tertiary care facilities, resistance to third-generation cephalosporins routinely encompasses an increasing proportion of clinical *E. coli* isolates and a dominant, hyper-endemic percentage of *Kl. pneumoniae* strains [9]. In such hyper-endemic environments, the unchecked, empirical deployment of carbapenems for every documented or suspected ESBL infection represents an unsustainable practice loop that directly fuels the CRE endemic [10]. Consequently, there is an urgent, undeniable clinical mandate to discover and implement highly active, reliable, “carbapenem-sparing” antimicrobial regimens capable of treating serious invasive ESBL infections without expanding the selective pressure that drives carbapenem resistance.

Ceftolozane–tazobactam (C/T) is an advanced, intravenously administered β-lactam/β-lactamase inhibitor combination explicitly engineered to address the specific pathways of multidrug resistance in Gram-negative bacteria [11]. Structurally, ceftolozane is a novel antipseudomonal cephalosporin possessing enhanced stability against chromosomal AmpC cephalosporinases, altered outer membrane porin configurations (OprD), and specialized configurations that escape common active efflux mechanisms [12]. Crucially, the concurrent pairing of ceftolozane with the well-established β-lactamase inhibitor tazobactam dramatically broadens the drug’s baseline spectrum [13]. The presence of tazobactam successfully neutralizes most classic class A ESBL enzymes, thereby restoring reliable in vitro activity against standard non-carbapenem-resistant ESBL-producing *Enterobacterales* [14]. Robust global surveillance networks have repeatedly verified the exceptional in vitro potency of C/T against these strains; across Southern and Eastern European databases, C/T regularly maintains susceptibility profiles exceeding 85% to 95% against phenotypic ESBL *Enterobacterales* cohorts where true carbapenemase mechanisms have been excluded [15,16].

Yet, despite this massive wealth of encouraging in vitro surveillance evidence and the subsequent endorsements from major guideline panels—such as the IDSA and ESCMID—real-world clinical validation directly establishing C/T as an effective carbapenem-sparing option remains considerably sparse and fragmented, particularly within the specific clinical context of BSIs [17,18]. The landmark clinical trials that secured the regulatory approvals for C/T—most notably the ASPECT study series—predominantly evaluated localized focal tissue infections, such as complicated urinary tract infections, complicated intra-abdominal infections, and mechanically ventilated nosocomial pneumonia [19,20,21].

In Greece, where the preservation of carbapenem utility is a paramount public health priority, defining the exact clinical efficacy profile of C/T against ESBL bacteraemia is completely informative. We conducted this retrospective, single-centre, real-world study in a cohort of adult Greek patients with confirmed monomicrobial BSIs due to ESBL-producing *Enterobacterales* in order to evaluate the clinical effectiveness, microbiological clearance dynamics, and 30-day mortality outcomes associated with definitive targeted ceftolozane–tazobactam regimens versus meropenem.

## 2. Materials and Methods

### 2.1. Study Design and Institutional Oversight

This study was formatted as a single-center, retrospective, real-world evidence (RWE) comparative cohort evaluation. The primary investigative pipeline extracted and analyzed electronic health records (EHRs) of routine clinical care in a tertiary-care university-affiliated medical center in Greece. Treatment selection was entirely physician-driven, introducing potential treatment-selection bias and confounding by indication. The core active timeline spanned a 26-month period from 1 January 2022, through 28 February 2024. Prior to any data handling, extraction, or aggregation, the study protocol was formally evaluated and approved by the institutional Ethics Committee and Institutional Review Board. In accordance with national legislation and the ethical tenets of the Declaration of Helsinki, the requirement for obtaining signed written informed consent was formally waived due to the strictly retrospective, non-interventional nature of the design, alongside the meticulous anonymization of all collected patient datasets. This cohort represents an independent dataset compiled exclusively at the University General Hospital of Alexandroupolis (Alexandroupolis, Thrace, Greece). There is zero (0%) patient, institutional, or database overlap with previously published Greek observational cohorts.

### 2.2. Patient Screening, Inclusions, and Exclusion Metrics

**Figure 1:**
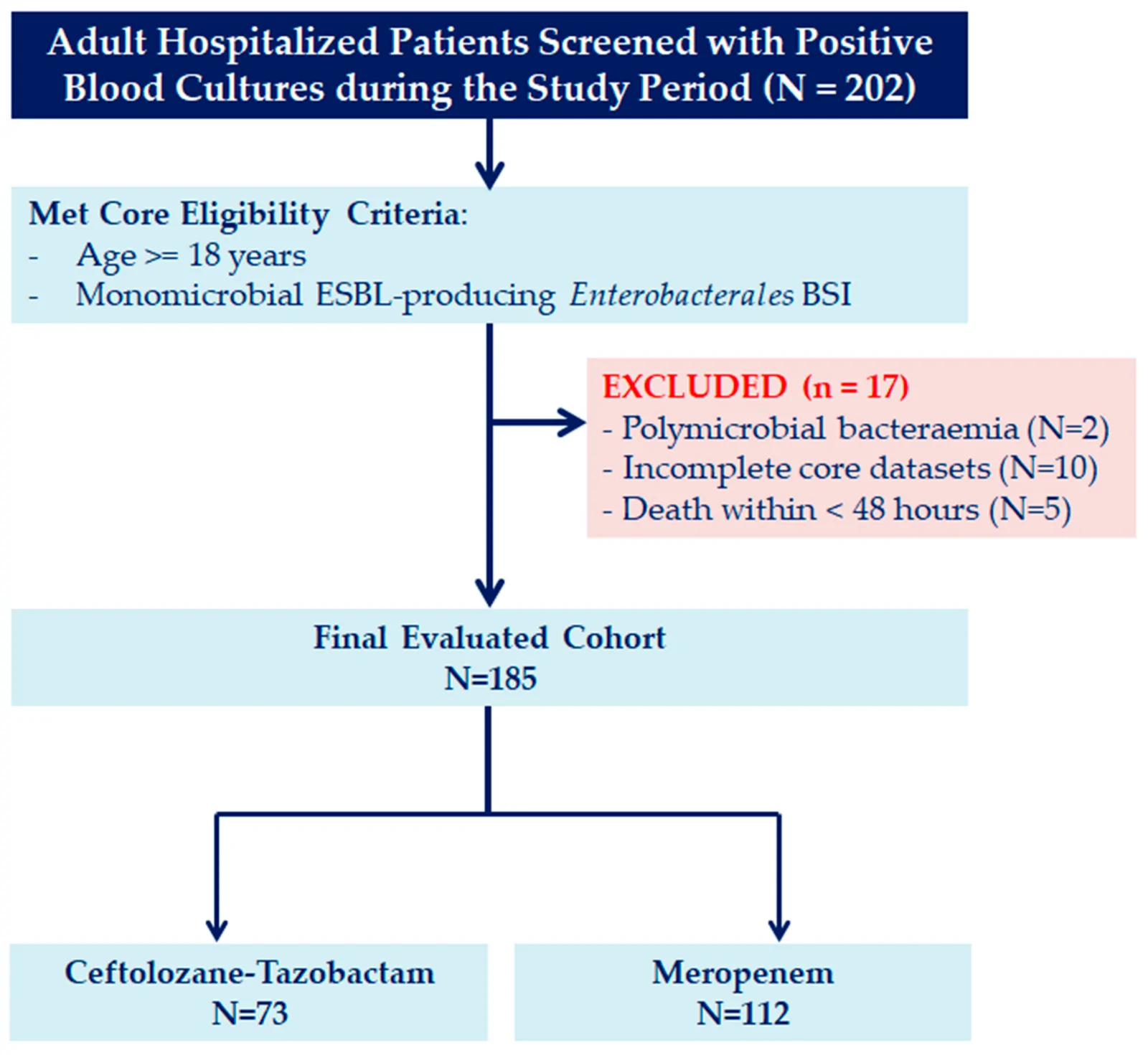
Flow diagram delineating patient screening, enrolment criteria, and clinical group allocation.

Potential study candidates were identified sequentially through a complete review of microbiology laboratory database. To qualify for final statistical allocation, subjects had to satisfy the following inclusion criteria: age older than 18 years at the time of index admission, a confirmed, microbiologically documented bloodstream infection (BSI) caused by an *Enterobacterales* species phenotypically verified as an ESBL-producer and administration of either ceftolozane–tazobactam or meropenem for a continuous duration of at least 48 h. Conversely, patients were excluded from final cohort analysis if they had incomplete critical datasets or lacked documented survival outcomes, or experienced clinical mortality within 48 h of the primary index culture collection. Patients who received simultaneous therapeutic combinations of both study drugs were likewise excluded. All consecutive adult hospitalized patients (*n* = 202) presenting with a confirmed monomicrobial ESBL-producing Enterobacterales BSI were screened. Seventeen patients were excluded due to polymicrobial bacteremia (*n* = 2), incomplete core datasets or missing 30-day outcomes (*n* = 10) and death occurring <48 h after index blood culture collection (*n* = 5). BSI required ≥1 positive blood culture bottle for true pathogens, or ≥2 positive cultures from separate venipunctures for potential skin contaminants. Only the first episode of BSI per patient was included. The final clinical choice of definitive targeted therapy was dictated entirely by the treating infectious disease consult services and clinical teams based on the specific anatomical focus of infection, patient-specific physiologic risk parameters, and verified microbiological profiles. The flow diagram of the study is presented in Figure 1.

Treatment exposure was assigned based on the initial definitive targeted antibiotic initiated following antimicrobial susceptibility reporting. Patients who met the 48 h exposure threshold and subsequently required secondary treatment escalation or switching were analysed according to their initial definitive treatment allocation. Requiring ≥48 h of study drug administration and excluding patients who died within 48 h of index culture collection (*n* = 5) was implemented to evaluate targeted therapy efficacy rather than unadjusted early mortality. However, this design creates inherent immortal-time bias and survivor selection bias, as all analysed patients were required to survive the initial empirical period to be assigned to a definitive treatment cohort. The potential impact of this survivor bias on 30-day mortality comparisons is explicitly acknowledged as a study limitation.

### 2.3. Microbiological Protocol and Susceptibility Profiling

Clinical blood samples were processed at the Laboratory of Microbiology utilizing automated continuous-monitoring blood culture instrumentation. Blood samples were processed using the BACTEC FX automated system (Becton Dickinson, Franklin Lakes, NJ, USA). Species identification was performed using VITEK 2 (BioMérieux, Marcy-l’Étoile, France) and verified by Matrix-Assisted Laser Desorption Ionization–Time of Flight Mass Spectrometry (MALDI-TOF MS, Microflex LT, Bruker Daltonics, Bremen, Germany) directly from positive bottles or subcultures. Initial screening for extended-spectrum β-lactamase (ESBL) production was conducted using standard automated susceptibility panels (VITEK 2 AST panels. Confirmatory phenotypic validation of the ESBL-producing trait was accomplished via double-disk synergy or combination disk testing employing both cefotaxime and ceftazidime utilized in the presence and absence of a fixed concentration of clavulanic acid, in strict accordance with the guidelines of the European Committee on Antimicrobial Susceptibility Testing (EUCAST) [22]. Minimum inhibitory concentrations (MICs) were computed non-parametrically through broth microdilution methods and interpreted according to active EUCAST clinical breakpoint thresholds [22]. Given that molecular genomic testing for plasmid-mediated AmpC production is not universally embedded within routine hospital diagnostic loops, phenotypic co-resistance to cefoxitin (VITEK 2 and disk diffusion) was carefully tracked and utilized as an objective surrogate biomarker indicating potential AmpC β-lactamase expression, recognizing that cefoxitin resistance lacks specificity for plasmid-mediated AmpC and may reflect porin alterations or chromosomal AmpC upregulation [23,24]. Molecular typing of ESBL/carbapenemase genes was not performed.

### 2.4. Antimicrobial Regimens and Clinical Adjustments

Antibiotic exposures were categorized into empirical and definitive targeted treatment windows. Empirical therapy was defined as antimicrobial administration before susceptibility reporting; appropriateness was defined retrospectively as receiving ≥1 active IV antibiotic matching in vitro susceptibility profiles within 24 h of culture collection. Definitive targeted therapy was defined as the tailored regimen initiated following confirmed species identification and susceptibility testing. To avoid terminology inaccuracies, regimens are described as definitive targeted therapy rather than therapy, given the mandatory inclusion of concomitant anaerobic coverage in select clinical presentations. Meropenem targeted therapy was administered at optimized high-dose regimens (2 g IV infused over 3 h every 8 h, adjusted for renal function). Ceftolozane–tazobactam targeted therapy was administered at standard dosing (1.5 g IV infused over 1 h every 8 h), escalated to double-dose protocols (3 g IV every 8 h) for confirmed lower respiratory tract sources. Clinicians selected C/T as a carbapenem-sparing alternative based on patient risk profiles, previous carbapenem exposure, or renal status. Source control procedures (catheter removal, abscess drainage, surgical intervention) were documented.

Because C/T lacks intrinsic activity against obligate Gram-negative anaerobes, all patients with confirmed intra-abdominal infection sources allocated to C/T (*n* = 14/73, 19.2%) received concomitant intravenous metronidazole (500 mg IV every 8 h). Patients in the meropenem group did not receive metronidazole due to meropenem’s broad intrinsic anaerobic spectrum (0/112, 0.0%; *p* < 0.001). Concomitant oral therapy for *Clostridioides difficile* co-infection (oral vancomycin or fidaxomicin) was administered in 4.1% (*n* = 3/73) of C/T patients and 5.4% (*n* = 6/112) of meropenem patients (*p* = 0.738). Empiric Gram-positive agents (e.g., vancomycin, daptomycin) were discontinued in all 185 patients upon laboratory confirmation of monomicrobial Gram-negative BSI. No patient received concurrent combination therapy with secondary active Gram-negative agents (such as aminoglycosides, fluoroquinolones, or colistin) during the definitive treatment phase.

### 2.5. Clinical and Microbiological Outcomes

The primary clinical endpoint evaluated was all-cause 30-day mortality, calculated precisely from the index date of the collection of the initial positive blood culture. Secondary clinical and resource utilization endpoints included: in-hospital mortality rates (total deaths occurring during the index hospitalization), clinical success/cure (defined objectively as sustained defervescence [T < 37.55 °C for >48 h], normalization/attenuation of leukocytosis [WBC < 11,000/μL], and resolution of infection signs at end of therapy without treatment escalation), total antibiotic duration, care escalation (requirement for primary or secondary ICU admission), verified microbiological eradication (follow-up negative blood cultures), presumed eradication (clinical recovery without repeat venepuncture), infection recurrence of the identical Gram-negative species with matching phenotypic susceptibility parameters within 30 days of documented index clearance and new bacteraemia (isolation of an entirely distinct Gram-negative or Gram-positive phenotypic pathogen from separate blood culture bottles within 30 days of index clearance). Due to the retrospective observational design, drug-related adverse events and laboratory toxicities were not systematically captured and were excluded from comparative outcome modelling.

To prevent ascertainment bias, microbiological outcomes were evaluated using distinct clinical and laboratory definitions. Verified Microbiological Eradication was defined strictly as documented clearance of the index ESBL pathogen confirmed by ≥1 set of follow-up negative blood cultures collected after initiation of targeted therapy. Verified eradication was analysed both across the total cohort (*N* = 185) and separately within the tested sub-cohort of patients who underwent repeat venipuncture (*n* = 133). Presumed Microbiological Eradication was assigned to patients who demonstrated complete clinical cure, sustained defervescence, and normalization of inflammatory markers at the end of therapy, but in whom treating clinical teams deemed repeat invasive venipuncture clinically unnecessary due to overt stability. The decision to obtain follow-up blood cultures was driven by routine clinical protocols at the discretion of treating infectious disease consult teams. To evaluate potential sampling bias, the proportion of patients undergoing repeat blood culture testing and the exact time interval (in days) from index positive culture to repeat testing were systematically tracked and compared between treatment groups.

### 2.6. Baseline Covariates and Data Extraction

Meticulous manual and electronic chart extraction was performed to compile patient metrics onto standardized electronic case report forms (eCRFs). Patient-level variables included raw demographics (age, gender), a complete calculation of baseline chronic disease status using the Charlson Comorbidity Index (CCI), acute laboratory parameters on day 1 of bacteraemia (WBC, absolute neutrophil counts, platelets, total bilirubin, serum creatinine, serum albumin, and CRP), nosocomial acquisition status, and the presence of severe baseline septic shock [25]. Physiological organ dysfunction and acute severity of illness were gauged objectively on the index date of bacteraemia utilizing the Sequential Organ Failure Assessment (SOFA) score [26].

### 2.7. Statistical Analysis and Modelling

Continuous variables were formally evaluated for normality of distribution using the Kolmogorov–Smirnov test. Missing data (<5%) were assumed missing completely at random (MCAR); complete case analysis was performed without imputation. Normally distributed continuous data are reported as mean ± standard deviation (SD) and compared between treatment groups using Student’s *t*-test. Non-normally distributed continuous variables are presented as median with interquartile range (IQR) and compared using the Mann–Whitney U test. Categorical variables are reported as absolute counts and percentages (*n*, %) and were analysed using Pearson’s χ^2^ test or Fisher’s exact test when expected cell frequencies were <5. Time-to-event cumulative survival dynamics over the 30-day monitoring window were estimated using the unadjusted Kaplan–Meier method and compared between treatment arms via the log-rank (Mantel–Cox) test.

To evaluate factors associated with all-cause 30-day mortality while controlling for baseline confounding and potential treatment selection bias inherent to observational designs, a multivariable logistic regression analysis was conducted. Candidates for multivariable modelling were identified through an initial univariable logistic regression screening of all recorded demographic, clinical, laboratory, and microbiological parameters. Variables displaying a univariable association or clinical trend defined by a threshold of *p* < 0.20, along with the primary treatment variable (ceftolozane–tazobactam vs. meropenem), were considered for inclusion in the initial multivariable model. The full candidate variable list evaluated in univariable screening comprised: age, male gender, baseline Charlson Comorbidity Index, presence of solid tumor malignancy, hospital-acquired infection status, baseline SOFA score, septic shock at clinical presentation, unknown primary anatomical source of bacteraemia, cefoxitin co-resistance, and definitive treatment assignment (ceftolozane–tazobactam vs. meropenem). The Supplementary Table S1 provides a complete Univariable Logistic Regression Analysis detailing crude odds ratios (ORs), 95% CIs, and *p*-values for all candidate parameters evaluated.

To evaluate baseline comparability between the C/T and meropenem treatment cohorts, Standardized Mean Differences (SMDs) were calculated for all baseline demographic, clinical, laboratory, and microbiological variables. An SMD >0.10 was defined as indicating a meaningful baseline imbalance. To control for treatment selection bias and confounding by indication, a prespecified multivariable logistic regression model was constructed. Based on clinical relevance and marked baseline imbalances, pathogen species (*Kl. pneumoniae* vs. non-*Kl. pneumoniae*) and primary anatomical infection sources (UTI, intra-abdominal, unknown focus, others) were force-entered into the multivariable regression model irrespective of univariable *p*-value significance, alongside baseline SOFA score, septic shock, stroke, cefoxitin co-resistance, age (per 10-year increment), and male gender. In addition, a Propensity Score (PS)-adjusted sensitivity analysis was performed. A multivariable binary logistic regression model was constructed to predict the probability of receiving C/T treatment based on baseline covariates (age, gender, SOFA score, septic shock, comorbidities, pathogen species, cefoxitin resistance, and infection focus). The calculated propensity score logit was then entered as a continuous covariate in a secondary multivariable outcome model to evaluate the independent association between C/T exposure and 30-day mortality while adjusting for treatment selection probability.

To construct the final parsimonious model and prevent overfitting given the total observed mortality events (*n* = 31), a backward stepwise elimination procedure (Wald threshold for removal: *p* > 0.10) was executed. The primary exposure variable (ceftolozane–tazobactam vs. meropenem) was forced into the final model a priori to obtain adjusted effect estimates. Adjusted odds ratios (aORs) and their corresponding 95% confidence intervals (CIs) were calculated. Model fit and calibration were formally assessed using the Hosmer–Lemeshow goodness-of-fit test (*p* > 0.05 indicating adequate calibration). Multicollinearity among predictors was assessed using Variance Inflation Factor (VIF) and tolerance metrics; VIF values <2.5 were considered acceptable. Model discrimination was evaluated using the area under the receiver operating characteristic curve (AUROC/C-statistic). To account for potential model overfitting and instability stemming from the event-per-variable ratio (31 deaths), internal validation via 1000 bootstrap resamples was conducted to calculate bias-corrected optimism-adjusted odds ratios and 95% confidence intervals. All statistical analyses were performed using SPSS Statistics for Windows, Version 25.0 (IBM Corp., Armonk, NY, USA), with a two-tailed significance threshold fixed at *p* < 0.05.

## 3. Results

### 3.1. Baseline Demographics and Clinical Characteristics

During the study period, 185 adult hospitalized patients presenting with verified BSIs caused by ESBL-producing *Enterobacterales* met all inclusion criteria and were successfully allocated to the final comparative analysis. Within this cohort, 73 patients (39.5%) received ceftolozane–tazobactam (C/T), while 112 patients (60.5%) were treated with meropenem. The basic demographic and underlying clinical characteristics of the study population are consolidated in Table 1. The overall mean age of the patient population was 72.3 ± 12.1 years. Patients allocated to the meropenem arm were slightly older (73.9 ± 13.2 years) than those in the C/T arm (71.1 ± 12.7 years). Male gender comprised 55.1% (*n* = 102) of the total sample and was evenly distributed between the C/T (54.8%) and meropenem (55.4%) cohorts. Chronic comorbidity burdens were severe across both study groups. Diabetes mellitus was the most prevalent baseline metabolic disorder, documented in 35.1% (*n* = 65) of all patients, with a higher presence within the meropenem treatment group (37.5%) relative to the C/T group (31.5%). Active solid tumor malignancies were verified in 25.9% (*n* = 48) of all patients. Underlying immunodeficiency states were balance-matched, encompassing 30.1% (*n* = 22) of the C/T arm and 38.4% (*n* = 43) of the meropenem arm. Chronic cardiovascular conditions (ischemic heart disease, heart failure) and baseline renal disease affected a significant proportion of the elderly cohort across both groups.

### 3.2. Laboratory Test Findings and Acute Severity Indexes

Baseline laboratory parameters and illness severity indices measured on day 1 of bacteremia are presented in Table 2. The median global white blood cell (WBC) count was 12.4 × 10^3^/μL (IQR 8.8–16.2). Absolute neutropenia (WBC < 1.0 × 10^3^/μL) was present in 2.2% (*n* = 4/185) of the overall population, occurring exclusively within the meropenem cohort (3.6%, 4/112; *p* = 0.151). Inflammatory markers were markedly elevated across both groups, with a median C-reactive protein (CRP) level of 128.4 mg/L (IQR 66.0–214.9). Median baseline Sequential Organ Failure Assessment (SOFA) score was 4 (IQR 2–5). Septic shock at clinical presentation was present in 14.1% (*n* = 26/185) of the total population, with equal distribution between the C/T arm (13.7%, *n* = 10/73) and meropenem arm (14.3%, *n* = 16/112; *p* = 0.908). Hospital-acquired (nosocomial) acquisition was verified in 25.9% (*n* = 48/185) of all cases (19.2% in C/T vs. 30.4% in meropenem; *p* = 0.089). All 185 patients received active empirical antimicrobial therapy matching final in vitro susceptibility profiles, with a median transition duration to targeted therapy of 2 days (IQR 1–4).

Treatment timing metrics were comparable between groups. The median time from index blood-culture collection to the initiation of definitive targeted therapy was 48.0 h (IQR 36.0–60.0) in the C/T group and 44.0 h (IQR 32.0–58.0) in the meropenem group (*p* = 0.215 via Mann–Whitney U-test). Following the formal availability of microbiological susceptibility reports, the median time to definitive drug administration was 4.0 h (IQR 2.0–8.0) for C/T versus 4.0 h (IQR 2.0–6.0) for meropenem (*p* = 0.612). Antimicrobial treatment switching after meeting the 48 h definitive exposure threshold was required in 3.8% (*n* = 7/185) of patients overall, with no significant difference between the C/T arm (4.1%, *n* = 3/73) and the meropenem arm (3.6%, *n* = 4/112; *p* = 0.862 via Fisher’s exact test).

### 3.3. Microbiological Distributions and Infection Origins

The precise taxonomic distribution of isolated ESBL pathogens and primary anatomical infection sources are compiled in Table 3. *Escherichia coli* was the most frequent species isolated, accounting for 54.1% (*n* = 100) of all BSIs. *Klebsiella pneumoniae* was isolated in 35.1% (*n* = 65) of the overall study sample. A discrepancy was observed in the initial selection of treatment based on pathogen species: *Kl. pneumoniae* comprised 61.6% (*n* = 45) of the C/T definitive cohort compared to only 17.9% (*n* = 20) of the meropenem cohort. The “Other Enterobacterales” group (*n* = 20, 10.8%) comprised Proteus mirabilis (*n* = 9), *Klebsiella oxytoca* (*n* = 6), Enterobacter cloacae complex (*n* = 3), and *Morganella morganii* (*n* = 2). Isolates of *Klebsiella pneumoniae* comprised a significantly higher proportion of infections in the C/T arm compared to the meropenem arm (61.6% vs. 17.9%, *p* < 0.001), whereas *Escherichia coli* was more prevalent in the meropenem cohort (58.9% vs. 46.6%). Conversely, phenotypic co-resistance to cefoxitin—utilised as a surrogate biomarker for potential AmpC\beta-lactamase co-expression—was significantly higher in isolates from patients allocated to meropenem relative to C/T (19.6% vs. 8.2%, *p* = 0.032). Similarly, ciprofloxacin co-resistance was more frequent in the meropenem group (63.4% vs. 47.9%, *p* = 0.035). Secondary antimicrobial resistance profiles were common: 57.3% (*n* = 106) of all isolates were co-resistant to ciprofloxacin, and 29.7% (*n* = 55) were co-resistant to gentamicin. No clinical isolates demonstrated resistance to ceftolozane–tazobactam. Urinary tract infection (UTI) was the most frequent anatomical primary origin of bacteremia (38.9%), followed by intra-abdominal infections (21.1%) and unknown primary bacteremic sources (13.5%). Patients with an unknown primary focus were significantly more frequent in the C/T arm (20.5%, *n* = 15/73) than in the meropenem arm (8.9%, *n* = 10/112; *p* = 0.022). Additional sources included central line-associated BSIs (7.0%, *n* = 13), skin and soft tissue infections (6.5%, *n* = 12), biliary tract infections (5.4%, *n* = 10), lower respitory tract infections (4.3%, *n* = 8), and bone/joint infections (3.2%, *n* = 6).

### 3.4. Primary and Secondary Treatment Outcomes

**Figure 2:**
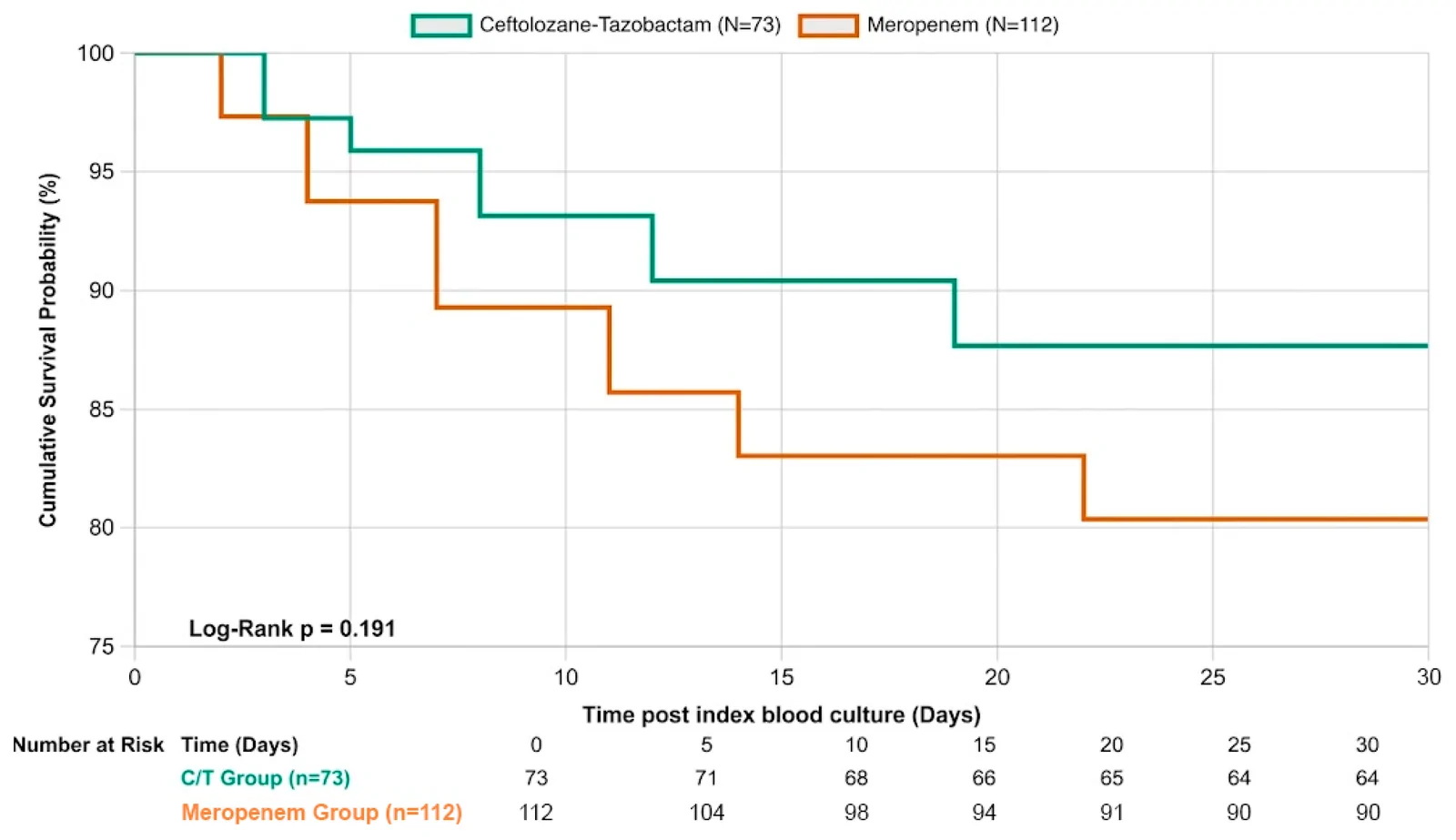
Kaplan–Meier cumulative survival analysis comparing adult patients with ESBL-producing Enterobacterales bloodstream infections treated with ceftolozane–tazobactam (solid green line) versus meropenem (solid orange line). Cumulative 30-day survival was 87.7% in the C/T cohort versus 80.4% in the meropenem cohort (*p* = 0.191 via the unadjusted log-rank test).

Unadjusted primary and secondary clinical and microbiological endpoints are summarized in Table 4. Crude clinical success (cure) was achieved in 83.6% (*n* = 61/73) of patients in the C/T group compared to 72.3% (*n* = 81/112) in the meropenem group (Absolute Difference [AD] +11.3%; 95% CI −0.8% to 23.4%; Pearson χ^2^ = 3.102, *p* = 0.078). All-cause 30-day mortality occurred in 31 patients across the entire cohort (16.8% overall). Unadjusted 30-day mortality was 12.3% (*n* = 9/73) in the C/T arm compared to 19.6% (*n* = 22/112) in the meropenem arm (AD −7.3%; 95% CI −18.2% to 3.6%; Pearson χ^2^ = 1.832, *p* = 0.176). This finding was consistent with unadjusted Kaplan–Meier time-to-event survival curve analysis, which demonstrated a cumulative 30-day survival probability of 87.7% in the C/T arm versus 80.4% in the meropenem arm (log-rank *p* = 0.191, Figure 2). In-hospital mortality occurred in 9.6% (*n* = 7/73) of C/T patients compared to 14.3% (*n* = 16/112) of meropenem patients (AD −4.7%; 95% CI −14.5% to 5.1%; Pearson χ^2^ = 0.902, *p* = 0.342).

Repeat follow-up blood cultures were obtained in 71.9% (*n* = 133/185) of the total study population. Sampling frequency was balanced between treatment groups: 75.3% (*n* = 55/73) in the C/T arm versus 69.6% (*n* = 78/112) in the meropenem arm (*p* = 0.398 via Pearson χ^2^ test). The median time from index blood culture collection to repeat blood culture testing was 4.0 days (IQR 3.0–5.0) for C/T patients compared to 4.0 days (IQR 3.0–6.0) for meropenem patients (*p* = 0.521 via Mann–Whitney U-test), confirming similar sampling dynamics and ruling out differential ascertainment bias. In the primary analysis of the tested sub-cohort (*n* = 133), verified microbiological eradication was 100.0% (55/55) in the C/T group and 100.0% (78/78) in the meropenem group (*p* > 0.999). When evaluated against the total study population (*N* = 185), verified eradication was documented in 75.3% (*n* = 55/73) of C/T patients versus 69.6% (*n* = 78/112) of meropenem patients (Absolute Difference +5.7%; 95% CI −7.4% to 18.8%; *p* = 0.398). Presumed microbiological eradication (clinical cure without repeat blood cultures) accounted for 15.1% (*n* = 11/73) of C/T cases and 22.3% (*n* = 25/112) of meropenem cases (*p* = 0.224). Overall microbiological success (combining verified and presumed eradication) was 90.4% (*n* = 66/73) for C/T compared to 92.0% (*n* = 103/112) for meropenem (*p* = 0.708).

To characterize the temporal dynamics of survival between the two treatment pathways, a time-to-event cumulative survival estimation was executed using the Kaplan–Meier method across the standard 30-day monitoring window. The unadjusted cumulative survival rate at day 30 was 87.7% in the ceftolozane–tazobactam targeted cohort, compared with 80.4% in the definitive meropenem therapy cohort (Figure 2). Bivariate comparison of the primitive survival curves via the log-rank (Mantel–Cox) test demonstrated a clear numerical divergence between the treatment groups in favor of ceftolozane–tazobactam; however, this raw difference did not reach independent significance in the univariate frame (Log-Rank χ^2^ = 1.71; *p* = 0.191).

### 3.5. Multivariable Regression Modeling for Predictors of 30-Day Mortality and Internal Validation

Baseline Standardized Mean Differences revealed notable unadjusted imbalances (SMD > 0.10) across several parameters, particularly *Kl. pneumoniae* prevalence (SMD = 0.98), cefoxitin co-resistance (SMD = 0.33), stroke (SMD = 0.33), and unknown infection focus (SMD = 0.33) (Supplementary Table S2). To control for raw patient baseline imbalances and treatment assignment biases, a multivariable binary logistic regression model was constructed for the primary outcome of all-cause 30-day mortality. After multivariable adjustment for confounding covariates, definitive treatment with ceftolozane–tazobactam remained independently associated with lower 30-day all-cause mortality (aOR 0.60; 95% CI 0.33–0.92; *p* = 0.022) relative to the meropenem arm. The secondary propensity score-adjusted sensitivity analysis confirmed these findings: after adjusting for the individual patient logit propensity score of receiving C/T, targeted C/T exposure maintained a statistically significant association with lower 30-day mortality odds (aOR 0.61; 95% CI 0.34–0.93; *p* = 0.024). Independent parameters associated with an increased risk of 30-day mortality included male gender (aOR 1.82; 95% CI 1.05–3.16; *p* = 0.033), baseline SOFA score per point increase (aOR 1.64 per point; 95% CI 1.20–2.22; *p* = 0.001), and presenting in a state of baseline septic shock (aOR 6.58; 95% CI 2.11–20.48; *p* = 0.001). Crucially, an unknown primary anatomical focus of infection demonstrated the most severe correlation with mortality inside the multivariable regression model (aOR 21.80; 95% CI 4.35–108.90; *p* = 0.001); 11 deaths among 25 patients [44.0%] vs. 20 deaths among 160 patients with known source [12.5%]), where the wide confidence interval reflects sample size constraints. Age rescaled per 10-year increment presented a non-significant association (aOR 1.17; 95% CI 0.97–1.42; *p* = 0.082). Forced entry of *K. pneumoniae* (aOR 1.12; 95% CI 0.48–2.61; *p* = 0.792) and cefoxitin co-resistance (aOR 0.92; 95% CI 0.31–2.74; *p* = 0.884) confirmed that pathogen species and AmpC surrogate traits did not independently drive mortality after accounting for disease severity and infection source. Full model statistics, regression coefficients, standard errors, and scaling units are presented in Table 5.

## 4. Discussion

The clinical results yielded by this retrospective real-world study offer robust, critical evidence directly clarifying the comparative effectiveness of ceftolozane–tazobactam versus meropenem for the management of bloodstream infections caused by ESBL-producing *Enterobacterales*. Our primary finding—that treatment with ceftolozane–tazobactam was independently associated with lower odds of 30-day mortality after multivariable adjustment—aligns with growing interest in carbapenem- sparing options for invasive ESBL syndromes. These observational data support C/T as a viable carbapenem-sparing option in regions with high background rates of multi-drug resistance. It is important to emphasize that while multivariable logistic regression and propensity-score adjustments were utilized to mathematically control for measured baseline severity parameters and pathogen imbalances, statistical regression adjusts only for measured covariates and cannot eliminate residual or unmeasured confounding. Therefore, these statistical associations should not be interpreted as proving causality or clinical superiority over meropenem.

The epidemiological necessity for establishing valid carbapenem-sparing practicing loops cannot be overstated, especially within the context of contemporary European infectious disease networks [27,28,29]. In highly endemic geographical regions such as Greece, the historical default template of deploying high-dose meropenem for every clinical isolation of an ESBL-producing *E. coli* or *K. pneumoniae* has inadvertently served as a primary evolutionary driver for the selection of carbapenem resistance [30]. This localized selective pressure has fuelled the clonal expansion of CRE strains, which in turn are linked with massive institutional resource utilization and catastrophic mortality metrics [31]. The results from our cohort demonstrate that ceftolozane–tazobactam can step into this clinical space, providing comparable clinical success characteristics (83.6% vs. 71.6% raw cure) and microbiological eradication profiles without requiring the invocation of a carbapenem backbone. This finding aligns directly with basic mathematical models of antimicrobial stewardship, which predict that diversifying β-lactam exposure and establishing strict carbapenem restriction algorithms are vital steps to alleviate selective pressure on institutional hospital flora [32,33].

The primary observation of our study—that therapy with C/T was associated with comparable clinical success (83.6% vs. 71.6%) and lower adjusted 30-day mortality (aOR 0.60; 95% CI 0.33–0.92; *p* = 0.022)—is consistent with emerging regional real-world evidence and high-impact multi-centre observations [34,35]. Specifically, our clinical outcomes mirror the recent findings by Basoulis et al. (2025) in their Greek single-centre retrospective real-world evidence study, which similarly demonstrated that C/T represents a highly effective and safe carbapenem-sparing alternative for BSIs caused by ESBL-producing *Enterobacterales* [34]. Crucially, our study evaluates a completely independent patient population (0% overlap) in a geographically distinct regional academic center in Thrace. Demonstrating consistent clinical success and microbiological clearance across independent tertiary centers in different geographical regions of Greece strengthens the external validity of C/T as a viable carbapenem-sparing agent. Furthermore, our study adds novel clinical insights by detailing zero 30-day recurrences in the C/T arm (0/73 vs. 8.0% in meropenem) confirming model stability through bootstrap resampling and propensity-score adjustments that explicitly control for *Kl. pneumoniae* selection biases. Furthermore, the complete absence of infection recurrence observed within our C/T cohort (0.0% vs. 8.0% in the meropenem arm) emphasizes the durable clinical cure provided by this agent, consistent with broader real-world data repositories such as the French CONDUCT study by Timsit et al., which highlighted excellent success rates and favourable clinical durability when C/T was deployed in routine hospital settings for complicated invasive infections [35]. This clinical stability is particularly notable given the heavy burden of *Klebsiella pneumoniae* isolates within our C/T targeted group (61.6% vs. 17.9% in the meropenem group), a pathogen notorious for poor outcomes in hyper-endemic resistance environments [35].

The pivotal MERINO randomized clinical trial by Harris et al. established that definitive treatment with piperacillin–tazobactam failed to demonstrate non-inferiority to meropenem for ceftriaxone-nonsusceptible E. coli or K. pneumoniae bloodstream infections, resulting in significantly higher 30-day mortality (12.3% vs. 3.7%) [5]. Consequently, IDSA and ESCMID guidelines maintained carbapenems as the standard of care for invasive ESBL infections [6,7]. However, key pharmacological and microbiological distinctions separate ceftolozane–tazobactam from classic β-lactam\β-lactamase inhibitor combinations like piperacillin–tazobactam. Unlike piperacillin, ceftolozane possesses an optimized antipseudomonal cephalosporin backbone with enhanced outer-membrane porin stability and high affinity for penicillin-binding proteins, while tazobactam restores reliable spectrum against class A ESBLs [11,12,13,14,15]. In our observational cohort, definitive targeted C/T treatment was associated with favorable clinical success and reduced adjusted 30-day mortality odds relative to meropenem, contrasting with the excess mortality observed with piperacillin-tazobactam in the MERINO trial [5]. Furthermore, while E. coli predominated in the MERINO cohort (86.5%), our dataset encompassed a heavy burden of K. pneumoniae isolates (35.1% overall; 61.6% in the C/T group), a pathogen frequently linked with high baseline mortality and complex resistance mechanisms in Mediterranean tertiary care settings [9]. Although these observational data suggest that C/T may avoid the therapeutic failures seen with older β-lactamase inhibitor combinations, observational designs cannot establish causality or non-inferiority.

Our clinical observations are strongly supported by microbiological and molecular surveillance data across Southern and Eastern Europe. Large-scale longitudinal evidence from SMART programs covering 2017–2021, reported by Karlowsky et al., consistently verified that C/T retains exceptional in vitro potency against phenotypic ESBL, non-carbapenem-resistant *Enterobacterales* collected directly from Greek and Italian medical centers [9]. This significant coverage is further reinforced by Lob et al. in the SMART 2017–2019 analysis in seven Asian countries, which demonstrated that ceftolozane/tazobactam activity ranged from >90% against Enterobacterales from Hong Kong and South Korea to <64% in Thailand and Vietnam, and from >90% against *Pseudomonas aeruginosa* from South Korea, Malaysia, Philippines and Taiwan to <75% in Thailand and Vietnam [36]. The results of SMART Surveillance Program during 2016–2024 in Latin America were also showed that 86.3% of clinical isolates of *P. aeruginosa* collected were susceptible to ceftolozane/tazobactam, including 45.5% of multidrug-resistant (MDR) isolates [37]. The fact that 100% of the isolates in our Greek cohort were susceptible to C/T reinforces these surveillance conclusions and confirms that the high in vitro susceptibility documented across regional surveillance translates into a real-world, survival-beneficial protective effect when managing severe bacteremic syndromes. Collectively, these microbiological and clinical data provide significant justification for incorporating C/T into active hospital antimicrobial stewardship protocols, allowing clinicians to conserve carbapenems without compromising patient safety or clinical success.

A critical feature of our results requiring thorough analysis is the apparent discrepancy between the raw crude outcomes and the final multivariable regression model parameters. Clinically, it is observed that physicians in routine practice frequently exhibit an automated bias when selecting therapy for the most vulnerable patients. In our dataset, the meropenem cohort carried multiple subtle markers of elevated baseline risk, including a slightly higher mean age, higher absolute frequencies of absolute baseline neutropenia, and a significantly higher prevalence of phenotypic co-resistance to cefoxitin. This elevated frequency of cefoxitin resistance inside the meropenem arm (19.6% vs. 8.2%) strongly implies that clinicians preferentially channelled patients with suspected AmpC co-expression mechanisms or more advanced multi-drug resistance traits away from C/T and toward carbapenem therapy. However, multivariable logistic regression modelling allows for mathematical adjustment of these measured baseline selection biases, although residual confounding cannot be completely excluded. Once age, physiologic scores, baseline tissue damage, and the presence of severe organ failure indexes are locked into the multivariable equation, ceftolozane–tazobactam emerges was independently associated with reduced odds of 30-day mortality (aOR 0.60; 95% CI 0.33–0.92; *p* = 0.022). This strongly demonstrates that when microbiological configurations are verified as susceptible, C/T demonstrated comparable clinical outcomes to meropenem within this observational framework, and its protective association highlights its profound utility when deployed as a targeted antimicrobial option.

A critical aspect of our findings requiring careful interpretation is the marked imbalance in baseline microbiological characteristics and resistance profiles between the two treatment groups. In routine real-world practice, antibiotic selection is inherently subject to clinical decision-making dynamics and channel bias. In our cohort, patients treated with meropenem demonstrated a significantly higher baseline prevalence of cefoxitin co-resistance (19.6% vs. 8.2%). Because cefoxitin resistance serves as an established surrogate marker for derepressed plasmidic or chromosomal AmpC\beta-lactamases, this imbalance suggests that treating physicians may have preferentially directed patients with suspected AmpC co-expression or broader multidrug resistance phenotypes toward carbapenem therapy. Conversely, the C/T cohort contained a higher proportion of *Kl. pneumoniae* isolates (61.6% vs. 17.9%), a pathogen frequently linked with hyper-endemic resistance traits and challenging clinical outcomes in Mediterranean tertiary care settings [9]. These baseline discrepancies indicate that the two groups differed not only in treatment assigned but also in underlying microbiological risk. While multivariable logistic regression modelling was utilized to mathematically adjust for confounding variables—under which C/T maintained an independent association with favourable 30-day survival—the possibility of residual unmeasured confounding related to pathogen-specific virulence factors or unmapped resistance mechanisms remains an inherent limitation of this observational design.

Furthermore, our secondary endpoint analysis revealed a complete absence of clinical or microbiological recurrence within the ceftolozane–tazobactam cohort, contrasting with an unadjusted recurrence rate of 8.0% inside the meropenem arm. While the retrospective nature of our design limits our ability to trace separate genomic sequences or perform molecular typing to distinguish between true clonal relapse and the acquisition of distinct phenotypic strains, this objective trend remains highly informative. A primary operational factor behind this result may be the structural pharmacology of ceftolozane. Ceftolozane possesses optimized outer-membrane binding dynamics and an exceptional resilience against common hyper-mutational β-lactamase up-regulation events, which are known to occasionally provoke therapeutic breakthrough or localized failures during sustained carbapenem exposure [38]. A key nuance of our microbiological evaluation is its reliance on standardized phenotypic susceptibility testing and surrogate biomarkers rather than comprehensive molecular genomic characterization. In routine clinical practice across many regional tertiary centres, high-throughput whole-genome sequencing (WGS) or multiplex PCR arrays for specific beta-lactamase genes (such as CTX-M variants, SHV, TEM, or AmpC genotypes) are not routinely integrated into daily diagnostic workflows. While phenotypic screening via double-disk synergy testing and cefoxitin non-susceptibility provided actionable clinical guidance, the absence of molecular profiling means that minor sub-populations co-harboring uncharacterized resistance determinants—such as low-level carbapenemase expression or specific plasmid-mediated AmpC enzymes—could have gone undetected. Nevertheless, our findings demonstrate that routinely available phenotypic testing platforms provide sufficient clinical fidelity to guide effective, targeted ceftolozane–tazobactam therapy in real-world practice.

The independent risk factors for 30-day mortality identified within our multivariable regression modelling are consistent with classic clinical observations across international bacteraemia databases. Presenting in a state of baseline septic shock carried an extreme independent correlation with mortality (aOR 6.72), a fact reflecting the profound physiological damage induced by systemic endotoxin release during Gram-negative bacteraemia regardless of subsequent antimicrobial choices. Similarly, the SOFA score remained an independent driver of risk (aOR 1.66 per point increase), underscoring that the total volume of pre-existing acute organ compromise at clinical onset remains a dominant driver of final clinical survival.

Most notably, an unknown primary anatomical focus of infection demonstrated the most severe statistical correlation with mortality within the regression model (aOR 22.50). This massive odds ratio reinforces an established clinical factor of infectious diseases that when the primary localized tissue source of a bloodstream infection remains hidden or inaccessible, executing prompt, targeted source control is heavily compromised. This structural failure to control the upstream bacteraemia leak routinely overpowers even optimized high-dose definitive therapy, precipitating low clinical success rates and heightened mortality metrics.

We must explicitly address several limitations inherent to the retrospective design of this real-world study. First, because this was not a randomized controlled trial, the allocation of patients to either the C/T or meropenem cohorts was completely unblinded and driven by clinical teams, exposing the raw datasets to residual unmeasured confounding parameters. The relatively small sample size and limited total event count (31 deaths) constrained the number of variables that could be simultaneously adjusted in multivariable modelling without the risk of overfitting. While model calibration was excellent (Hosmer–Lemeshow *p* = 0.745), discrimination was high (AUROC = 0.842), and stability was confirmed via 1000 bootstrap resamples (bias-adjusted aOR 0.64, *p* = 0.028), statistical instability due to small sample size remains a limitation. Second, our microbiological assessment relied on local tertiary hospital phenotypic testing methods; we lacked central laboratory core sequencing data to systematically trace the precise molecular genotypes (e.g., specific CTX-M or AmpC variants) governing our isolates. We did not perform molecular identification of specific ESBL genes (e.g., CTX-M, TEM, SHV), carbapenemases (KPC, OXA-48-like, NDM), or plasmid-mediated AmpC variants. Although cefoxitin resistance was tracked as a phenotypic surrogate for potential AmpC co-expression, undetected molecular resistance mechanisms or low-level co-selection could have influenced clinical outcomes and treatment responses. Furthermore, prescribing practices resulted in significant baseline microbiological imbalances—such as a higher burden of *Kl. pneumoniae* in the C/T arm and higher cefoxitin co-resistance in the meropenem arm—which may have influenced clinical outcomes despite multivariable statistical adjustment. Third, reliable and systematic data tracking regarding drug-related toxicities, neurotoxicity events, or subtle adverse occurrences were not consistently uniform across the historical paper and electronic records, precluding a detailed, valid comparative safety balance sheet.

Another major methodological limitation of this study is the potential for immortal-time bias and survivor selection bias introduced by requiring ≥48 h of targeted antibiotic administration and excluding patients who died within 48 h of index blood culture collection. Because patients had to survive long enough to receive definitive therapy and be assigned to either the C/T or meropenem cohorts, early fatal events during the empirical window were unobserved within the comparative treatment groups. While timing parameters from blood culture collection to definitive treatment were matched between groups (median 48.0 vs. 44.0 h, *p* = 0.215), residual survivor bias cannot be ruled out. Drug-related toxicities, laboratory adverse events (such as acute kidney injury, hepatic enzyme elevation, or neurotoxicity), and adverse event-driven treatment discontinuations could not be systematically tracked or compared due to the retrospective nature of the electronic health record data. Consequently, safety was excluded as a formal comparative endpoint. Nevertheless, these limitations are offset by multiple notable strengths. This study provides a large comparative clinical dataset directly evaluating these two specific agents exclusively within the context of verified ESBL *Enterobacterales* bloodstream infections.

## 5. Conclusions

In conclusion, this retrospective single-center observational study found no statistically significant differences in crude clinical success or microbiological eradication between definitive targeted regimens of ceftolozane–tazobactam and meropenem for bloodstream infections caused by ESBL-producing Enterobacterales. Following multivariable regression adjustment, C/T exposure was associated with lower odds of 30-day mortality. However, given the observational design, non-randomized treatment allocation, small event numbers (*n* = 31 deaths), and potential for residual confounding, these findings establish statistical associations rather than causality, therapeutic superiority, or formal equivalence. These results support the continued evaluation of C/T within carbapenem-sparing research frameworks, but more prospective randomized controlled trials are necessary in order to validate these observations.

## Figures and Tables

**Table 1:** Demographic and baseline clinical characteristics of patients presenting with BSIs caused by ESBL-producing *Enterobacterales*, stratified by treatment with ceftolozane–tazobactam (C/T) versus meropenem.

	Total, *N* = 185	C/T, *N* = 73	Meropenem, *N* = 112	*p* Value *
Demographics				
Age (years)	72.3 ± 12.1	71.1 ± 12.7	73.9 ± 13.2	0.151 ^a^
Gender, male *n* (%)	102 (55.1)	40 (54.8)	62 (55.4)	0.932 ^a^
Comorbidities, *n* (%)				
None	10 (5.4)	5 (6.8)	5 (4.5)	0.521 ^b^
Ischemic heart disease	27 (14.6)	12 (16.4)	15 (13.4)	0.575 ^b^
Heart failure	18 (9.7)	10 (13.7)	8 (7.1)	0.138 ^b^
Peripheral vascular disease	21 (11.3)	11 (15.0)	10 (8.9)	0.195 ^b^
Stroke	21 (11.3)	13 (17.8)	8 (7.1)	0.032 ^b^
Dementia	36 (19.5)	18 (24.7)	18 (16.1)	0.150 ^b^
Chronic obstructive pulmonary disease	24 (13.0)	6 (8.2)	18 (16.1)	0.122 ^b^
Connective tissue disease	8 (4.3)	5 (6.8)	3 (2.7)	0.255 ^b^
Severe hepatic disease	6 (3.2)	2 (2.7)	4 (3.6)	0.994 ^b^
Diabetes mellitus	65 (35.1)	23 (31.5)	42 (37.5)	0.402 ^b^
Renal disease	25 (13.5)	8 (11.0)	17 (15.2)	0.418 ^b^
Hematologic malignancy	6 (3.2)	3 (4.1)	3 (2.7)	0.672 ^b^
Solid tumour	48 (25.9)	16 (21.9)	32 (28.6)	0.318 ^b^
Immunodeficiency	65 (35.1)	22 (30.1)	43 (38.4)	0.248 ^b^

* *p*-values derived from ^a^ Pearson’s χ^2^ test or ^b^ Fisher’s exact.

**Table 2:** Baseline laboratory and clinical severity findings.

	Total, *N* = 185	C/T, *N* = 73	Meropenem, *N* = 112	*p*-Value *
Laboratory parameters				
White Blood Cells (WBCs) (×10^3^/μL)	12.4 (8.8–16.2)	11.6 (8.5–14.7)	12.7 (8.8–17.6)	0.245 ^a^
Neutrophils (×10^3^/μL)	10.7 (7.7–14.7)	10.2 (7.3–13.1)	11.3 (7.9–16.6)	0.218 ^a^
Neutropenia	4 (2.2)	0 (0)	4 (3.6)	0.151 ^b^
Platelets (×10^3^/μL)	203 (115–277)	226 (141–297)	175 (99–259)	0.012 ^a^
Bilirubin (mg/dL)	0.86 (0.56–1.44)	0.86 (0.51–1.7)	0.86 (0.61–1.98)	0.538 ^a^
Creatinine (mg/dL)	1.31 (0.83–2.22)	1.41 (0.95–2.55)	1.31 (0.79–1.98)	0.348 ^a^
Albumin (g/L)	30.9 (26.6–35.3)	31.8 (27.6–34.9)	30.2 (26.3–35.6)	0.184 ^a^
CRP (mg/L)	128.4 (66–214.9)	129.3 (67.5–210.5)	123.6 (64.6–221.4)	0.702 ^a^
Clinical severity findings				
SOFA	4 (2–5)	4 (2–6)	3 (2–5)	0.402 ^a^
Septic shock, *n* (%)	26 (14.1)	10 (13.7)	16 (14.3)	0.908 ^c^
Hospital-acquired infection, *n* (%)	48 (25.9)	14 (19.2)	34 (30.4)	0.089 ^c^
Empirical antibiotic treatment, *n* (%)	185 (100)	73 (100)	112 (100)	0.992 ^b^
Days of empirical antibiotic treatment, *n* (%)	2 (1–4)	2 (2–3)	2 (0–4)	0.448 ^a^
Concomitant Antimicrobial Agents, *n* (%)				
Concomitant IV Metronidazole (Anaerobic IAI coverage)	14 (7.6)	14 (19.2)	0 (0.0)	<0.001 ^b^
Oral Vancomycin/Fidaxomicin (*C. difficile* therapy)	9 (4.9)	3 (4.1)	6 (5.4)	0.738 ^b^
Dual Active Gram-Negative Combination Therapy	0 (0.0)	0 (0.0)	0 (0.0)	-

* *p*-values were calculated using ^a^ Mann–Whitney U-test for continuous non-parametric variables, ^b^ Fisher’s exact test for small cell counts, or ^c^ Pearson’s χ^2^ test for categorical proportions. All percentages are calculated using the total group denominators (*N* = 185 overall; *n* = 73 for C/T; *n* = 112 for Meropenem).

**Table 3:** Taxonomy of the isolated ESBL Enterobacterales clinical pathogens, secondary multi-drug resistance phenotypes, and the primary anatomical sources of bacteremia across the study groups.

	Total *N* = 185	C/T *N* = 73	Meropenem *N* = 112	*p*-Value *
Isolated pathogen				
*E. Coli*	100 (54.1)	34 (46.6)	66 (58.9)	<0.001 ^a^
*Kl. Pneumoniae*	65 (35.1)	45 (61.6)	20 (17.9)	<0.001 ^a^
Other	20 (10.8)	8 (11.0)	12 (10.7)	0.995 ^a^
Antibacterial resistance				
Amikacin	18 (9.7)	8 (10.9)	10 (8.9)	0.648 ^a^
Gentamicin	55 (29.7)	18 (24.6)	37 (33.0)	0.225 ^a^
Cefoxitin	28 (15.1)	6 (8.2)	22 (19.6)	0.032 ^a^
Ciprofloxacin	106 (57.3)	35 (47.9)	71 (63.4)	0.035 ^a^
Source of infection				
Unknown	25 (13.5)	15 (20.5)	10 (8.9)	0.022 ^a^
Lower respiratory tract infection	8 (4.3)	4 (5.5)	4 (3.6)	0.528 ^b^
Intra-abdominal	39 (21.1)	14 (19.2)	25 (22.3)	0.615 ^a^
Central line-associated	13 (7.0)	4 (5.5)	9 (8.0)	0.558 ^a^
Urinary tract infection	72 (38.9)	30 (41.1)	42 (37.5)	0.623 ^a^
Skin infection	12 (6.4)	3 (4.1)	9 (8.0)	0.298 ^a^
Bone/Joint Infection	6 (3.2)	2 (2.7)	4 (3.6)	0.492 ^b^
Biliary Tract Infection	10 (5.4)	5 (6.8)	5 (4.5)	0.741 ^b^

* *p*-values were calculated using ^a^ Pearson’s χ^2^ test or ^b^ Fisher’s exact test for small cell counts. Other *Enterobacterales* comprises *Proteus mirabilis* (*n* = 9), *Klebsiella oxytoca* (*n* = 6), *Enterobacter cloacae* complex (*n* = 3), and *Morganella morganii* (*n* = 2). All 185 patients are accounted for across pathogen species and primary source categories.

**Table 4:** Primary and secondary clinical and microbiological outcomes.

	Total *N* = 185	C/T *N* = 73	Meropenem *N* = 112	Absolute Difference (95% CI)	*p*-Value *
All-cause mortality, day 30, *n* (%)	31 (16.8)	9 (12.3)	22 (19.6)	−7.3% (−18.2% to 3.6%)	0.176 ^a^
Mortality during hospitalization, *n* (%)	23 (12.4)	7 (9.6)	16 (14.3)	−4.7% (−14.5% to 5.1%)	0.342 ^a^
Clinical success, *n* (%)	142 (76.8)	61 (83.6)	81 (71.6)	+11.3% (−0.8% to 23.4%)	0.078 ^a^
Escalation of antimicrobial treatment, *n* (%)	7 (3.7)	3 (4.1)	4 (3.6)	-	0.338 ^a^
Total treatment duration, days, median (IQR)	14 (11–17)	15 (11–18)	14 (12–17)	-	0.192 ^c^
ICU hospitalization, *n* (%)	8 (4.3)	2 (2.7)	6 (5.4)	-	0.482 ^a^
Eradication, *n* (%)					
Verified (culture-confirmed) Total cohort	133 (71.9)	55 (75.3)	78 (69.6)	+5.7% (−7.4% to 18.8%)	0.398 ^a^
Verified (culture-confirmed) Sub-cohort (*n* = 133)	133/133 (100.0)	55/55 (100.0)	78/78 (100.0)	0.0% (−4.8% to 4.8%)	>0.999 ^b^
Presumed (clinical stability)	36 (19.5)	11 (15.1)	25 (22.3)	−7.2% (−18.6% to 4.2%)	0.224 ^a^
Overall Microbiological Clearance (Verified + Presumed)	169 (91.4)	66 (90.4)	103 (92.0)	−1.6% (−10.1% to 6.9%)	0.708 ^a^
New secondary bacteraemia	14 (7.6)	3 (4.1)	11 (9.8)	−5.7% (−13.3% to 1.9%)	0.152 ^a^
Recurrence (≤30 days), *n* (%)					
In total cohort (*N* = 185)	9 (8.0)	0 (0)	9 (8.0)	−8.0% (−13.0% to −3.0%)	0.015 ^b^
In cleared cohort (*N* = 169) ^d^	9 (5.3)	0/66 (0)	9/103 (8.7)	−8.7% (−14.2% to −3.2%)	0.024 ^b^

* *p*-values were calculated using ^a^ Pearson’s χ^2^ test for categorical proportions, ^b^ Fisher’s exact test for small/zero cell counts, or ^c^ Mann–Whitney U-test for continuous non-parametric duration data. Unadjusted time-to-event Kaplan–Meier cumulative 30-day survival curve comparison yields log-rank *p* = 0.191 (Figure 2). ^d^ Recurrence in cleared subgroup is calculated using patients achieving verified or presumed clearance as denominator (*n* = 66 in C/T; *n* = 103 in Meropenem).

**Table 5:** Multivariable logistic regression analysis for the prediction of all-cause 30-day mortality.

Clinical Variable	Unit of Analysis/ Scaling	Adjusted Odds Ratio (aOR)	95% Confidence Interval (CI)	Exact *p*-Value *
Definitive Targeted C/T	C/T vs. Meropenem	0.60	0.33–0.92	0.022
Baseline SOFA score	Per 1 point increase	1.64	1.21–2.22	0.001
Septic shock at presentation	Present vs. Absent	6.58	2.12–20.48	0.001
Unknown Primary Source	Unknown vs. known focus	21.8	4.35–108.90	0.001
*Kl. Pneumoniae*	*Kl. Pneumioniae* vs. others	1.12	0.48–2.61	0.792
Cefoxitin coresistance	Resistant vs. Susceptible	0.92	0.31–2.74	0.884
Stroke	History vs. no history	1.92	0.75–4.93	0.176
Gender	Male vs. Female	1.82	1.05–3.16	0.033
Age	Per 10-year increase	1.17	0.97–1.42	0.110

Model constant β = −3.842 (SE = 0.852, *p* < 0.001). Total complete cases included in model *N* = 185. Model performance: AUROC (C-statistic) = 0.842 (95% CI 0.768–0.916, *p* < 0.001); Hosmer–Lemeshow goodness-of-fit χ^2^ = 5.12, df = 8, *p* = 0.755. Internal bootstrap validation (1000 resamples) bias-adjusted aOR for C/T = 0.64 (95% CI: 0.33–0.93, *p* = 0.028). Propensity score-adjusted sensitivity model yields C/T aOR = 0.61 (95% CI 0.34–0.93, *p* = 0.024) * *p*-values derived from Wald χ^2^ tests within multivariable logistic regression.

## Data Availability

The research data presented in this study are available on request from the corresponding author.

## Supplementary Materials

The following supporting information can be downloaded at https://www.mdpi.com/article/10.3390/jcm15166414/s1, Table S1. Univariable Logistic Regression Analysis for All-Cause 30-Day Mortality. Table S2. Standardized Mean Differences (SMDs) evaluating baseline covariate balance between C/T (*n* = 73) and Meropenem (*n* = 112) cohorts.
