# Supplementary material for: Comparison of Ceftolozane–Tazobactam Versus Meropenem Regimens in Treating Bloodstream Infections Caused by Extended-Spectrum β-Lactamase-Producing Enterobacterales: Real-World Data from a Greek Tertiary Center
Source: J Clin Med. 2026 Aug 19;15(16):6414. doi: 10.3390/jcm15166414 (PMC13513874; doi:10.3390/jcm15166414)
Supplement: Supplementary file 1 [file jcm-15-06414-s001.zip › jcm-4442775-supplementary.pdf]

Supplementary Table S1. Univariable Logistic Regression Analysis for All-Cause 30-Day Mortality

Variable	Crude Odds Ratio (cOR)	95% Confidence Interval (CI)	p-value	Selected for Multivariable Model?
**Ceftolozane-Tazobactam (vs. Meropenem)**	0.57	0.25 – 1.32	0.188	Yes (p < 0.20 clinical focus)
**Age** (per year)	1.04	1.00 – 1.08	0.048	Yes (p<0.20)
**Male Gender**	1.72	0.78 – 3.79	0.178	Yes (p<0.20)
**Baseline SOFA Score** (per point)	1.48	1.21 – 1.81	< 0.001	Yes (p<0.20)
**Septic Shock at Presentation**	5.21	2.18 – 12.44	< 0.001	Yes (p<0.20)
**Unknown Primary Source**	8.40	3.12 – 22.61	< 0.001	Yes (p<0.20)
**Charlson Comorbidity Index**	1.15	0.96 – 1.38	0.125	No (Replaced by specific SOFA/Stroke parameters)
**Solid Tumor Malignancy**	1.42	0.62 – 3.25	0.408	No (p≥ 0.20)
**Hospital-Acquired Infection**	1.35	0.61 – 2.98	0.456	No (p≥ 0.20)
**Cefoxitin Co-resistance**	1.18	0.42 – 3.31	0.755	Yes (Forced entry; AmpC surrogate)
**Stroke**	2.81	1.07-7.34	0.035	Yes (p<0.20)
**Klebsiella pneumoniae**	2.45	1.12-5.36	0.024	Yes (Forced entry; baseline imbalance)

Supplementary Table S2. Standardized Mean Differences (SMDs) evaluating baseline covariate balance between C/T (n=73) and Meropenem (n=112) cohorts.

Baseline Variable	C/T Group (n=73)	Meropenem Group (n=112)	Unadjusted Standardized Mean Difference (SMD)	Imbalance Present? (SMD>0.10)
**Age (years), mean ±SD**	71.1 ±12.7	73.9 ±13.2	0.22	Yes
**Male Gender, n (%)**	40 (54.8%)	62 (55.4%)	0.01	No
**Stroke, n (%)**	13 (17.8%)	8 (7.1%)	0.33	Yes
**Diabetes Mellitus, n (%)**	23 (31.5%)	42 (37.5%)	0.13	Yes
**Baseline SOFA Score, median (IQR)**	4 (2–6)	3 (2–5)	0.12	Yes
**Septic Shock at Presentation, n (%)**	10 (13.7%)	16 (14.3%)	0.02	No
***Klebsiella pneumoniae*, n (%)**	45 (61.6%)	20 (17.9%)	0.98	Yes (Major)
***Escherichia coli*, n (%)**	34 (46.6%)	66 (58.9%)	0.25	Yes
**Cefoxitin Co-resistance, n (%)**	6 (8.2%)	22 (19.6%)	0.33	Yes
**Ciprofloxacin Resistance, n (%)**	35 (47.9%)	71 (63.4%)	0.31	Yes
**Urinary Source, n (%)**	30 (41.1%)	42 (37.5%)	0.07	No
**Unknown Primary Focus, n (%)**	15 (20.5%)	10 (8.9%)	0.33	Yes
